# Prevention of unplanned extubation in neonatal patients: Protocol for a systematic review and meta-analysis

**DOI:** 10.1371/journal.pone.0314201

**Published:** 2025-01-09

**Authors:** Ludmylla Cristina de Faria Pontes, Isac Davidson Santiago Fernandes Pimenta, Gidyenne Christine Bandeira Silva de Medeiros, Daniel Guillén-Martínez, Paloma Echevarria-Pérez, Grasiela Piuvezam, Helaine Carneiro Capucho

**Affiliations:** 1 Faculty of Health Sciences, Postgraduation Program in Health Sciences, University of Brasilia, Brasilia, Brazil; 2 Postgraduation Program in Public Health, Federal University of Rio Grande do Norte, Natal, Brazil; 3 Systematic Review and Meta-Analysis Laboratory (Lab-SYS), CNPq-UFRN, Natal, Brazil; 4 Faculty of Nursing, San Antonio de Murcia Catholic University, Murcia, Spain; Bahir Dar University College of Medical and Health Sciences, ETHIOPIA

## Abstract

Unplanned extubation (UPE), defined as accidental removal of the endotracheal tube during mechanical ventilation or its replacement due to suspected obstruction or inadequate diameter, is considered the fourth most common adverse event in neonatal intensive care units (NICU). This study aimed to describe a systematic review and meta-analysis protocol that will identify and assess the effect of primary intervention measures designed to prevent UPE in NICU. A search will be carried out in the following databases: PubMed/Medline, EMBASE, Scopus, CINAHL, Cochrane Library, SciELO, and LILACS. Reviewers, in pairs and independently, will select the studies, perform data extraction and assess the methodological quality of the included studies using preestablished tools according to the type of study. The systematic review will provide evidence to present the main intervention measures used in the prevention of UPE during the care of critical neonatal patients. The systematic review and meta-analysis resulting from this protocol may provide important information regarding UPE in the neonatal population, which will help with decision-making and the implementation of safer clinical practices that focus on the reduction of adverse events, contributing to the improvement of service management and the safety of neonatal patients.

## Introduction

The use of invasive procedures, such as endotracheal intubation for mechanical ventilation, is often essential in neonatal care. While these interventions are critical for survival, they come with significant risks, particularly for neonates with severe and complex health conditions. Prolonged intubation can increase the likelihood of adverse events, leading to extended hospitalization and heightened risks of morbidity and mortality [1,2].

One of the most prevalent adverse events in neonatal intensive care units (NICUs) is unplanned extubation (UPE), characterized by accidental removal of the endotracheal tube (ETT) during mechanical ventilation or its replacement due to suspected obstruction or inadequate diameter [1]. The incidence of UPE ranges from 0.56 to 5.3 per 100 days of intubation [1,3]. The consequences of this event can be detrimental to neonatal patients, including trauma to the larynx and trachea, hypoxia, hypercapnia, barotrauma, pneumothorax, increased risk of infection, and peri-intraventricular haemorrhage. Furthermore, UPE prolongs both hospital stay and the duration of mechanical ventilation, significantly compromising patient safety and quality of care provided and incurring substantial healthcare costs [1,4–9].

Several factors contribute to the occurrence of UPE in neonates, including skin immaturity, extended periods of intubation, and limited use of sedation, all of which hinder endotracheal tube stabilization [1]. Nevertheless, even in critically ill neonatal patients, UPE can be prevented. The implementation of programs or initiatives aimed at improving processes has emerged as a pivotal strategy for mitigating the risk of UPE in the NICU, and may include activities such as educational programs, stricter monitoring, device fixation among other initiatives [3,10].

Despite the importance of adopting preventive measures to mitigate UPE, systematic reviews have only explored the risk factors related to its occurrence, not the effectiveness of such interventions [1,11]. Therefore, the objective of this study is to describe a systematic review and meta-analysis protocol to identify and assess the effect of interventions to prevent UPE in neonatal intensive care units.

## Methodology

### Study registration

This systematic review protocol was registered in the International Prospective Register of Systematic Reviews (PROSPERO) under the registration number CRD42021265636. The protocol development followed the guidelines of the Cochrane Handbook for Systematic Reviews of Interventions and the Preferred Reporting Items for Systematic Reviews and Meta-Analyses Protocols (PRISMA-P) [12,13] (S1 File). As this is a systematic review protocol, submission and approval from a research ethics committee or institutional review board was not necessary.

### Research question

Our review aims to address the following research question: What is the effect of preventive measures designed to minimize the occurrence of unplanned extubations in the Neonatal Intensive Care Unit (NICU)?

### Eligibility criteria

#### Participants

This study will include newborn patients admitted to the NICU who require mechanical ventilation through an endotracheal tube. We will not impose any restrictions on the type of NICU or the profile of intubated neonatal patients receiving invasive mechanical ventilation (such as weight, gestational age, or reason for admission).

#### Intervention

The focus of this study will be on interventions aimed at reducing unplanned extubation in the neonatal ICU. We will consider all types of interventions, including changes in the endotracheal tube (ETT) fixation model, routine assessment of fixation material integrity and ETT positioning, and modifications in care routines or procedures. Interventions can be implemented individually or in combination, involving a single professional group or multidisciplinary team, and can have varying durations and frequencies.

#### Comparison

The comparison group will consist of newborn patients admitted to the NICU who have not undergone any measures aimed at preventing unplanned extubation. We will also consider studies without a control group or that compare two types of interventions.

#### Outcomes

The primary outcome of interest is the occurrence of unplanned extubation in the NICU. Additionally, we will consider other outcomes, such as hemodynamic instability, difficulty in reintubation after unplanned extubation, and increased length of hospital stay or use of mechanical ventilation. We will also consider adverse effects related to the intervention, such as skin damage and lesions.

#### Types of studies

We will include randomized or nonrandomized clinical trials, quasi-experimental studies with a control group, before-and-after studies without a control group, or interrupted time series.

#### Exclusion criteria

We will exclude studies that combined neonatal patients to pediatric and/or adult patients without proper separation of the results for each group. We will also exclude studies conducted in the transit to NICU (e.g., during intra- and interhospital transport).

### Search strategy and information sources

We will conduct searches in the following databases: PubMed/Medline, Excerpta Medica (EMBASE), Scopus, Cumulative Index to Nursing and Allied Health Literature (CINAHL), Cochrane Library, Scientific Electronic Library Online (SciELO), and Latin American Literature in Health Sciences (LILACS).

The search strategy will be adapted for each of the databases using controlled vocabulary MeSH (Airway Extubation; Infant, Newborn; Intensive care units, neonatal) and EMTREE (extubation; newborn; neonatal intensive care unit), as well as their respective entry terms. Uncontrolled vocabularies will also be used (unplanned extubation, accidental extubation, self-extubation, unintentional extubation, unexpected extubation, inadvertent extubation, unintended extubation, spontaneous extubation, and airway accident), which were identified in studies related to the subject. The search strategy for the PubMed/Medline is available in the S2 File.

We will not impose any restrictions on the language or publication period of the studies. If studies are published in languages unfamiliar to the reviewers, we will utilize the translation services of our institutions to translate the papers for assessment. We aim to complete the searches by August 2025.

### Study selection

We will export the records obtained through the search strategy to Rayyan® software for an initial check and removal of duplicate entries.

At least two reviewers will independently assess the titles and abstracts of each study in pairs. The full texts of potentially eligible studies will then be read by at least two reviewers, also in pairs and independently, to evaluate them based on the eligibility criteria. All reviewers will undergo prior training in study selection. Disagreements regarding inclusion criteria will be resolved by a third reviewer.

After reviewing the full texts, primary studies that do not meet the inclusion criteria will be excluded, with reasons for exclusion documented. We will also manually review the references of the included studies to identify any additional relevant studies not captured in the main search.

### Data extraction

At least two reviewers will independently extract the data in pairs using a previously tested standard electronic spreadsheet. The extracted data will include study characteristics (main author, year of publication, study country, study design, sample size), population details (gestational age, weight, total number of patient-days intubated, duration of mechanical ventilation), interventions employed (changes in routine care or procedures, standardization and surveillance of the endotracheal tube (ETT) fixation model, and positioning in the patient’s trachea), outcomes obtained (incidence of unplanned extubations (UPE), and associated complications such as hemodynamic instability and difficulty in reintubation). We expect to conclude the data extraction until January 2026.

#### Dealing with missing data

If data are missing or unclear, the research team will attempt to contact the corresponding author or co-author by email. If this communication is unsuccessful, we will exclude the from the analysis, and this will be addressed in the discussion section.

### Data synthesis

We will present the data in summary tables in a narrative form to describe the characteristics of the included studies. Studies with dichotomous results will be summarized by considering risk measures (odds ratio, relative risk, etc.) with their respective confidence intervals (95% CI). Continuous quantitative results were summarized as the mean differences and their respective confidence intervals (95% CI). The p values of the included measures will also be considered.

A meta-analysis will be conducted in cases in which the studies included in the systematic review were sufficiently similar. For this, the heterogeneity between the studies will be evaluated using chi-square statistics, with a significance value of p<0.05. I^2^ statistics will also be calculated to assess the inconsistency between studies. An I^2^ value of 0% to 40% indicates that the heterogeneity might not be important; 30% to 60% may represent moderate heterogeneity; 50% to 90% may represent substantial heterogeneity; 75% to 100%: considerable heterogeneity [12].

As we expect a considerable heterogeneity between the studies, a random effects model will be used [12]. If possible, funnel plots were used to assess the presence of possible publication biases, and a linear regression approach was used to assess plot asymmetry.

#### Subgroup analysis

If possible, we will conduct subgroup analysis based on type of interventions, risk of bias, study type (randomized and non-randomized), and patient profile, as gestational age, reason for admission, and birth weight (<1000g –extremely low birth weight; <1500g very low birth weight; and <2500g low birth weight).

To the risk of bias classification, we will consider a subgroup of the studies only with low risk of bias in the general classification.

### Risk of bias and quality assessment

Two reviewers will independently assess the risk of bias. The reviewers will receive prior training, and we will calculate the agreement between them using the kappa index. The tools will be employed according to study type: Revised Cochrane risk-of-bias tool for randomized trials (ROB 2.0) for randomized clinical trials; and Risk of Bias In Nonrandomized Studies of interventions (ROBINS-I) for nonrandomized and quasi-experimental studies [14–16].

We will use the Quality Assessment Tool for Before-After (Pre-Post) Studies with No Control Group, developed by the National Heart, Lung, and Blood Institute, to evaluate the before and after (Pre-Post) studies with no control group and interrupted time series studies [17].

We will also use the Grading of Recommendations, Evaluation, Development, and Analysis (GRADE) to assess the certainty of the evidence provided by the selected studies [18].

## Discussion

Unplanned extubation (UPE) has become a significant concern, instigating the identification of its causes and outcomes. Sharek et al. highlighted UPE as the fourth most common adverse event in neonatal intensive care units (NICUs), with many cases resulting in permanent damage [2]. Therefore, is essential to prevent this harmful event in neonatal patients.

Studies show that inadequate fixation of the endotracheal tube (ETT), patient movement, excessive secretions, nursing overtime hours, and prolonged mechanical ventilation (over 10.5 days) are linked to a higher UPE rate [5,19,20]. Strategies such as professional training, monitoring and more robust fixation techniques for ETTs may significantly reduce UPE in neonates [9,10,21,22].

Despite the importance of preventive measures to avoid this event in the NICUs, current systematic reviews have focused only on identifying risk factors for unplanned extubation in NICUs [1,11,23].

### Implications

Given the multifactorial causes of UPE, preventing its occurrence presents unique challenges for NICU teams [24]. UPE can lead to iatrogenic injuries and is an important indicator of care quality. Improving quality of care requires process improvements and the adoption of evidence-based best practices [25,26].

This systematic review and meta-analysis protocol aim to fill a significant gap in the literature by evaluating ways to prevent unplanned extubation (UPE) in neonatal intensive care units (NICUs). The findings are expected to provide solid evidence that healthcare professionals can use to make better clinical decisions, thereby enhancing patient safety and care quality in neonatal units. This study could also influence training programs, protocols, and policies. Ultimately, it highlights a commitment to reducing adverse events and ensuring high-quality care for neonates.

### Limitations

The systematic review and meta-analysis resulting from this protocol may have limitations in methodological quality, heterogeneity of results, and data availability from the identified studies. Including before and after studies and quasi-experimental studies, means incorporate the quality improvement projects into the review, that may not meet the necessary criteria for experimental studies [27–29].

Conducting experimental studies to evaluate the effectiveness of interventions in quality improvement settings can be logistically challenging and expensive. Additionally, ethical considerations prohibit the exclusion of critically ill and vulnerable neonatal patients from interventions that are considered superior to current practices [30,31]. However, the rigorous methodology applied in the review process, along with the careful interpretation of results based on the methodological quality of the included studies, can help address these limitations.

## Supporting information

S1 File PRISMA-P checklist. (DOC)

S2 File PubMed/Medline search strategy. (DOCX)
