# Supplementary material for: Prevention of unplanned extubation in neonatal patients: Protocol for a systematic review and meta-analysis
Source: PLoS One. 2025 Jan 9;20(1):e0314201. doi: 10.1371/journal.pone.0314201 (PMC11717187; doi:10.1371/journal.pone.0314201)
Supplement: S2 File — (DOCX) [file pone.0314201.s002.docx]

**S2 File. PubMed/Medline Search Strategy**

**#1** ("Airway Extubation"[MeSH Terms] OR "Airway Extubation"[All Fields] OR "Tracheal Extubation"[All Fields] OR "Tracheal Extubations"[All Fields] OR "Intratracheal Extubation"[All Fields] OR "Endotracheal Extubation"[All Fields] OR "Endotracheal Extubations"[All Fields] OR "Extubation"[Title/Abstract] OR "unplanned extubation"[All Fields] OR "accidental extubation"[All Fields] OR "self extubation"[All Fields] OR "unintentional extubation"[All Fields] OR "unexpected extubation"[All Fields] OR "inadvertent extubation"[All Fields] OR "unintended extubation"[All Fields] OR "spontaneous extubation"[All Fields] OR "airway accident"[All Fields])

**#2** ("infant, newborn"[MeSH Terms] OR "Newborn Infant"[All Fields] OR "Newborn Infants"[All Fields] OR "Newborns"[All Fields] OR "Newborn"[Title/Abstract] OR "Neonate"[Title/Abstract] OR "Neonates"[All Fields] OR "Neonatal"[Title/Abstract] OR "Postmature"[All Fields] OR "Premature"[Title/Abstract] OR "Preterm"[All Fields] OR "Prematurity"[All Fields] OR "intensive care units, neonatal"[MeSH Terms] OR "Neonatal Intensive Care Unit"[All Fields] OR "Newborn Intensive Care Unit"[All Fields] OR "Newborn Intensive Care Units"[All Fields] OR "Neonatal Intensive Care Units"[All Fields] OR "Neonatal ICU"[All Fields] OR "Newborn ICU"[All Fields] OR "Newborn ICUs"[All Fields] OR "baby"[All Fields] OR "babies"[All Fields])

**#3** = **#1** AND **#2**
